# Supplementary material for: Association between Sugar Intake and Intima Media Thickness as a Marker for Atherosclerosis: A Cross-Sectional Study in the Malmö Diet and Cancer Study (Sweden)
Source: Nutrients. 2021 May 5;13(5):1555. doi: 10.3390/nu13051555 (PMC8147969; doi:10.3390/nu13051555)
Supplement: Supplementary file 1 [file nutrients-13-01555-s001.zip › nutrients-1181979-supplementary.pdf]

Table S1. Association between free sugar intake (percentage on non-alcoholic energy intake, %E) and intima media thickness (mm) measured at the common carotid artery (IMTcca) and at the bifurcation of the carotids (IMTbif). IMT presented as mean value (95% confidence interval).

		Free sugar intake (%E)						
		**< 5**	**5 - 7.5**	**7.5 – 10**	**10 – 15**	**15 – 20**	**> 20**	**P_{trends}**
**IMTcca**								
N (men / women)*		*130 / 170*	*326 / 417*	*460 / 685*	*751 / 1224*	*220 / 368*	*63 / 104*	
Model 1	Men	0.766 (0.738 – 0.795)	0.760 (0.741 – 0.778)	0.762 (0.747 – 0.777)	0.756 (0.744 – 0.768)	0.753 (0. 731 – 0.775)	0.789 (0.749 – 0.829)	0.716
	Women	0.713 (0.694 – 0.732)	0.721 (0.709 – 0.733)	0.725 (0.715 – 0.734)	0.717 (0.710 – 0.725)	0.725 (0.712 – 0.738)	0.745 (0.721 – 0.769)	0.443
Model 2	Men	0.757 (0.727 – 0.787)	0.754 (0.733 – 0.774)	0.763 (0.746 – 0.780)	0.758 (0.744 – 0.772)	0.756 (0.732 – 0.780)	0.792 (0.751 – 0.834)	0.604
	Women	0.712 (0.692 – 0.732)	0.721 (0.708 – 0.735)	0.724 (0.713 – 0.735)	0.716 (0.707 – 0.726)	0.723 (0.708 – 0.737)	0.739 (0.713 – 0.765)	0.755
Model 3	Men	0.749 (0.717 – 0.782)	0.746 (0.724 – 0.768)	0.752 (0.732 – 0.772)	0.751 (0.734 – 0.767)	0.744 (0.719 – 0.770)	0.781 (0.738 – 0.824)	0.647
	Women	0.712 (0.692 – 0.733)	0.723 (0.708 – 0.737)	0.724 (0.712 – 0.736)	0.715 (0.705 – 0.726)	0.721 (0.706 – 0.736)	0.735 (0.709 – 0.761)	0.989
**IMTbif**								
N (men / women)*		*81 / 105*	*229 / 272*	*319 / 454*	*552 / 819*	*147 / 274*	*48 / 70*	
Model 1	Men	1.585 (1.451 – 1.719)	1.579 (1.489 – 1.660)	1.499 (1.431 – 1.568)	1.493 (1.440 – 1.545)	1.485 (1.385 – 1.586)	1.434 (1.260 – 1.608)	0.707
	Women	1.390 (1.285 – 1.494)	1.383 (1.318 – 1.449)	1.381 (1.330 – 1.431)	1.358 (1.320 – 1.396)	1.391 (1.326 – 1.456)	1.429 (1.301 – 1.557)	0.539
Model 2	Men	1.561 (1.416 – 1.707)	1.552 (1.462 – 1.643)	1.476 (1.397 – 1.555)	1.472 (1.410 – 1.534)	1.480 (1.372 – 1.589)	1.420 (1.240 – 1.600)	0.129
	Women	1.381 (1.270 – 1.491)	1.398 (1.325 – 1.471)	1.397 (1.338 – 1.455)	1.384 (1.336 – 1.432)	1.408 (1.335 – 1.482)	1.426 (1.288 – 1.564)	0.609
Model 3	Men	1.556 (1.399 – 1.712)	1.528 (1.427 – 1.628)	1.453 (1.363 – 1.544)	1.460 (1.386 – 1.533)	1.462 (1.346 – 1.579)	1.395 (1.207 – 1.582)	0.158
	Women	1.393 (1.279 – 1.508)	1.414 (1.336 – 1.492)	1.398 (1.332 – 1.463)	1.389 (1.333 – 1.445)	1.401 (1.324 – 1.479)	1.415 (1.276 – 1.554)	0.360

N: number of observations. *Due to the lack of information regarding certain covariates in Model 3, the number of observations (N) is lower for Model 3, i.e., for IMTcca N = 125, 318, 446, 726, 212 and 62 for men and N = 167, 411, 665, 1191, 359 and 104 for women and for IMTbif N = 77, 223, 312, 534, 141 and 48 for men and N = 102, 272, 439, 804, 269 and 70 for women. **Model 1:** Adjusted for age, start date, time between baseline and IMT measurement, season. **Model 2:** Adjusted for Model 1 plus alcohol consumption, leisure time – physical activity, education, smoking habits, BMI, energy intake, coffee, meat, fruits & vegetables, fibre, saturated fat. **Model 3:** Adjusted for Model 2 plus high triglycerides, low HDLc, high LDLc and hypertension. Statistical significance established for $P<0.05$.

Table S2. Association between total sugar intake (percentage on non-alcoholic energy intake, %E) and intima media thickness (mm) measured at the common carotid artery (IMTcca) and at the bifurcation of the carotids (IMTbif). IMT presented as mean value (95% confidence interval).

		Total sugar intake (%E)				
		**< 15**	**15 - 20**	**20 – 25**	**> 25**	**P_{trends}**
**IMTcca**						
N (men / women)*		*423 / 249*	*800 / 909*	*513 / 1143*	*214 / 667*	
Model 1	Men	0.763 (0.747 – 0.779)	0.762 (0.750 – 0.774)	0.755 (0.740 – 0.769)	0.755 (0.733 – 0.778)	0.359
	Women	0.730 (0.714 – 0.745)	0.720 (0.712 – 0.728)	0.718 (0.710 – 0.725)	0.726 (0.716 – 0.735)	0.792
Model 2	Men	0.753 (0.733 – 0.772)	0.762 (0.748 – 0.776)	0.760 (0.744 – 0.777)	0.764 (0.740 – 0.789)	0.571
	Women	0.729 (0.711 – 0.747)	0.720 (0.710 – 0.730)	0.716 (0.707 – 0.725)	0.724 (0.712 – 0.736)	0.719
Model 3	Men	0.744 (0.722 – 0.766)	0.754 (0.737 – 0.771)	0.750 (0.732 – 0.769)	0.753 (0.727 – 0.779)	0.711
	Women	0.730 (0.711 – 0.748)	0.720 (0.708 – 0.731)	0.715 (0.704 – 0.726)	0.722 (0.709 – 0.735)	0.539
**IMTbif**						
N (men / women)*		*293 / 165*	*575 / 619*	*357 / 769*	*151 / 441*	
Model 1	Men	1.564 (1.493 – 1.636)	1.513 (1.462 – 1.565)	1.476 (1.411 – 1.651)	1.481 (1.382 – 1.579)	0.066
	Women	1.437 (1.353 – 1.521)	1.345 (1.301 – 1.388)	1.389 (1.350 – 1.428)	1.371 (1.320 – 1.422)	0.734
Model 2	Men	1.519 (1.432 – 1.607)	1.484 (1.421 – 1.548)	1.471 (1.397 – 1.546)	1.483 (1.374 – 1.591)	0.515
	Women	1.423 (1.329 – 1.517)	1.364 (1.311 – 1.417)	1.408 (1.360 – 1.457)	1.394 (1.331 – 1.457)	0.992
Model 3	Men	1.493 (1.394 – 1.592)	1.471 (1.394 – 1.547)	1.458 (1.373 – 1.543)	1.461 (1.345 – 1.577)	0.610
	Women	1.441 (1.343 – 1.538)	1.373 (1.313 – 1.433)	1.409 (1.352 – 1.467)	1.395 (1.326 – 1.464)	0.719

N: number of observations. *Due to the lack of information regarding certain covariates in Model 3, the number of observations (N) is lower for Model 3, i.e., for IMTcca N = 409, 781, 488 and 211 for men and N = 245, 885, 1115 and 652 for women and for IMTbif N = 281, 564, 340 and 150 for men and N = 163, 603, 755 and 435 for women. **Model 1:** Adjusted for age, start date, time between baseline and IMT measurement, season. **Model 2:** Adjusted for Model 1 plus alcohol consumption, leisure time – physical activity, education, smoking habits, BMI, energy intake, coffee, meat, fruits & vegetables, fibre, saturated fat. **Model 3:** Adjusted for Model 2 plus high triglycerides, low HDLc, high LDLc and hypertension. Statistical significance established for P<0.05.

Table S3. Association between treats intake (servings per week, svg/wk) and intima media thickness (mm) measured at the common carotid artery (IMTcca) and at the bifurcation of the carotids (IMTbif). IMT presented as mean value (95% confidence interval).

		Treats intake (svg/wk)					
		≤ 2	> 2 – 5	> 5 – 8	> 8 – 14	> 14	P_{trends}
**IMTcca**							
N (men / women)*		*243 / 261*	*490 / 859*	*449 / 790*	*532 / 785*	*236 / 273*	
Model 1	Men	0.758 (0.737 – 0.779)	0.759 (0.744 – 0.773)	0.768 (0.753 – 0.784)	0.749 (0.734 – 0.763)	0.770 (0.749 – 0.791)	0.912
	Women	0.721 (0.705 – 0.736)	0.720 (0.711 – 0.728)	0.722 (0.713 – 0.731)	0.719 (0.710 – 0.728)	0.729 (0.714 – 0.744)	0.642
Model 2	Men	0.758 (0.736 – 0.781)	0.763 (0.746 – 0.780)	0.770 (0.752 – 0.787)	0.749 (0.733 – 0.765)	0.764 (0.740 – 0.787)	0.681
	Women	0.721 (0.704 – 0.737)	0.719 (0.709 – 0.729)	0.722 (0.711 – 0.732)	0.718 (0.707 – 0.729)	0.726 (0.708 – 0.743)	0.960
Model 3	Men	0.752 (0.727 – 0.777)	0.755 (0.736 – 0.774)	0.762 (0.742 - 0.782)	0.737 (0.719 – 0.756)	0.753 (0.729 – 0.778)	0.398
	Women	0.722 (0.704 – 0.739)	0.718 (0.707 – 0.730)	0.721 (0.710 – 0.733)	0.717 (0.705 – 0.729)	0.726 (0.708 – 0.745)	0.902
**IMTbif**							
N (men / women)*		*162 / 173*	*356 / 555*	*326 / 542*	*369 / 542*	*163 / 182*	
Model 1	Men	1.602 (1.507 – 1.697)	1.490 (1.425 – 1.555)	1.508 (1.440 – 1.576)	1.506 (1.442 – 1.569)	1.481 (1.386 – 1.576)	0.210
	Women	1.381 (1.300 – 1.463)	1.397 (1.352 – 1.443)	1.356 (1.310 – 1.403)	1.367 (1.320 – 1.413)	1.384 (1.304 – 1.464)	0.548
Model 2	Men	1.553 (1.451 – 1.656)	1.466 (1.390 – 1.542)	1.490 (1.413 – 1.567)	1.489 (1.417 – 1.561)	1.454 (1.348 – 1.559)	0.442
	Women	1.387 (1.299 – 1.475)	1.415 (1.361 – 1.470)	1.377 (1.323 – 1.432)	1.389 (1.333 – 1.445)	1.390 (1.299 – 1.481)	0.515
Model 3	Men	1.538 (1.422 – 1.654)	1.455 (1.370 – 1.540)	1.478 (1.389 – 1.567)	1.476 (1.393 – 1.559)	1.427 (1.313 – 1.541)	0.361
	Women	1.393 (1.301 – 1.484)	1.414 (1.352 – 1.476)	1.388 (1.326 – 1.450)	1.396 (1.333 – 1.459)	1.386 (1.290 – 1.483)	0.511

N: number of observations. *Due to the lack of information regarding certain covariates in Model 3, the number of observations (N) is lower for Model 3, i.e., for IMTcca N = 232, 484, 432, 515 and 226 for men and N = 257, 842, 768, 769 and 261 for women and for IMTbif N = 155, 353, 314, 356 and 157 for men and N = 171, 543, 530, 536 and 176 for women. **Model 1:** Adjusted for age, start date, time between baseline and IMT measurement, season. **Model 2:** Adjusted for Model 1 plus alcohol consumption, leisure time – physical activity, education, smoking habits, BMI, energy intake, coffee, meat, fruits & vegetables, fibre, saturated fat. **Model 3:** Adjusted for Model 2 plus high triglycerides, low HDLc, high LDLc and hypertension. Statistical significance established for $P<0.05$.

Table S4. Association between topping intake (servings per week, svg/wk) and intima media thickness (mm) measured at the common carotid artery (IMTcca) and at the bifurcation of the carotids (IMTbif). IMT presented as mean value (95% confidence interval).

		Topping intake (svg/wk)					
		≤ 2	> 2 –7	> 7–14	> 14 – 28	> 28	P_{trends}
**IMTcca**							
N (men / women)*		*343 / 771*	*576 / 1276*	*451 / 583*	*368 / 253*	*212 / 85*	
Model 1	Men	0.775 (0.758 – 0.793)	0.749 (0.735 – 0.763)	0.756 (0.741 – 0.772)	0.768 (0.751 – 0.785)	0.754 (0.732 – 0.776)	0.776
	Women	0.725 (0.716 – 0.734)	0.718 (0.711 – 0.725)	0.719 (0.708 – 0.729)	0.721 (0.706 – 0.737)	0.746 (0.719 – 0.772)	0.744
Model 2	Men	0.774 (0.754 – 0.794)	0.753 (0.737 – 0.769)	0.760 (0.743 – 0.777)	0.770 (0.751 – 0.789)	0.742 (0.718 – 0.767)	0.408
	Women	0.725 (0.714 – 0.735)	0.717 (0.708 – 0.726)	0.717 (0.705 – 0.729)	0.718 (0.701 – 0.735)	0.739 (0.711 – 0.768)	0.847
Model 3	Men	0.765 (0.743 – 0.788)	0.744 (0.726 – 0.763)	0.752 (0.733 – 0.772)	0.761 (0.741 – 0.782)	0.729 (0.703 – 0.755)	0.347
	Women	0.725 (0.713 – 0.737)	0.717 (0.707 – 0.727)	0.714 (0.701 – 0.727)	0.719 (0.701 – 0.736)	0.738 (0.710 – 0.767)	0.738
**IMTbif**							
N (men / women)*		*232 / 515*	*407 / 851*	*312 / 396*	*264 / 168*	*161 / 64*	
Model 1	Men	1.577 (1.497 – 1.657)	1.510 (1.449 – 1.571)	1.496 (1.427 – 1.565)	1.463 (1.388 – 1.539)	1.521 (1.425 – 1.616)	0.288
	Women	1.400 (1.353 – 1.448)	1.353 (1.315 – 1.448)	1.382 (1.328 – 1.436)	1.334 (1.251 – 1.416)	1.541 (1.407 – 1.675)	0.811
Model 2	Men	1.551 (1.460 – 1.643)	1.505 (1.434 – 1.476)	1.492 (1.415 – 1.569)	1.430 (1.346 – 1.514)	1.443 (1.335 – 1.551)	0.096
	Women	1.418 (1.361 - 1.475)	1.374 (1.327 – 1.421)	1.400 (1.337 – 1.462)	1.336 (1.246 – 1.426)	1.490 (1.349 – 1.631)	0.586
Model 3	Men	1.534 (1.430 – 1.638)	1.486 (1.403 – 1.570)	1.481 (1.392 – 1.570)	1.420 (1.327 – 1.512)	1.431 (1.315 – 1.546)	0.186
	Women	1.426 (1.361 – 1.491)	1.379 (1.324 – 1.433)	1.398 (1.329 – 1.466)	1.350 (1.256 – 1.444)	1.493 (1.352 – 1.635)	0.578

N: number of observations. *Due to the lack of information regarding certain covariates in Model 3, the number of observations (N) is lower for Model 3, i.e., for IMTcca N = 326, 563, 436, 357 and 207 for men and N = 756, 1244, 568, 245 and 84 for women and for IMTbif N = 220, 399, 299, 258 and 159 for men and N = 506, 832, 389, 165 and 64 for women. **Model 1:** Adjusted for age, start date, time between baseline and IMT measurement, season. **Model 2:** Adjusted for Model 1 plus alcohol consumption, leisure time – physical activity, education, smoking habits, BMI, energy intake, coffee, meat, fruits & vegetables, fibre, saturated fat. **Model 3:** Adjusted for Model 2 plus high triglycerides, low HDLc, high LDLc and hypertension. Statistical significance established for $P<0.05$.
